# A pilot study to determine the feasibility of collecting amniotic fluid samples from women during labour and measuring amniotic fluid lactate at point of care

**DOI:** 10.1186/1756-0500-6-112

**Published:** 2013-03-26

**Authors:** Beverley Hall, Jenna Iwasenko, Mary Moriatis, William D Rawlinson, Mark B Tracy, Sally K Tracy

**Affiliations:** 1 University of Sydney, Darlington, NSW 2006, Australia; 2 Midwifery and Women’s Health Nursing Research Unit, Royal Hospital for Women, Randwick, NSW 2031, Australia; 3 Virology Research, Department of Microbiology, Prince of Wales Hospital, Randwick, NSW 2031, Australia; 4 School of Medical Sciences, Faculty of Medicine, University of New South Wales, Kensington, NSW 2031, Australia; 5 Department of Clinical Chemistry, South Eastern Area Laboratory Services, Randwick 2031, Australia; 6 Centre for Newborn Care, Westmead Hospital, Westmead, NSW 2145, Australia

**Keywords:** Dystocia, Lactate, Amniotic fluid, Labour, Point of care

## Abstract

**Background:**

The level of lactate in amniotic fluid may provide useful clinical information when assessing progress of a woman’s labour and if so, a rapid, reliable method to assess amniotic fluid lactate is required in order to be clinically relevant. However, measuring lactate levels in amniotic fluid, using portable, handheld lactate meters may be less accurate than reference laboratory instruments designed to measure lactate levels in aqueous solutions. Prior to conducting a large study, we assessed recruitment, consent and sampling procedures, and the accuracy of a handheld lactate meter to measure lactate in amniotic fluid. We compared amniotic fluid lactate results obtained using the hand held Lactate Pro (Arkray) to results obtained using reference laboratory methods ABX Pentra 400 (Horiba).

**Results:**

We recruited 35 nulliparous women during their antenatal hospital visits and tested amniotic fluid samples collected from 20 labouring women. The handheld Lactate Pro meter was found accurate from 9–20 mmol/L with a Passing & Bablok regression of y = 0.18 + 0.97x (95% CI 0.76–1.45). Amniotic fluid lactate results remained reliable in the presence of potential contaminants commonly encountered during labour; obstetric lubricant, blood and meconium.

**Conclusion:**

The measurement of amniotic fluid lactate using the Lactate Pro meter was reliable compared to reference laboratory methods for measuring lactate levels in amniotic fluid. The pilot study enabled the refinement of information, recruitment, consenting and sampling procedures prior to commencing a large cohort study.

## Background

Lack of progress in labour (dystocia) is one of the most commonly occurring problems requiring intervention during labour [1]. In many industrialized countries labours are treated for dystocia by augmenting with oxytocin to accelerate or improve progress [2,3]. However there is currently no accurate method for predicting at the onset of augmentation who will progress to a vaginal birth or who will require a cesarean section. In clinical practice, many labours deemed lacking in progress result in a normal vaginal birth. Conversely, women augmented for slow progress in labour may experience long painful labours that ultimately result in caesarean section.

Currently dystocia is diagnosed clinically by observing factors such as slowing of cervical dilation, slowing of descent and rotation of the fetal head, and changes in strength, duration and frequency of uterine contractions [4]. In the absence of a clear and consistent definition or marker to guide decision making there is considerable variation in diagnosis and clinical practice [5]. There is a need for improved diagnostic methods and decision making tools in the diagnosis of dystocia in order to reduce the high rates of labour intervention attributable to this condition.

Measurement of lactate in amniotic fluid of labouring women may be a surrogate biochemical marker for labour dystocia [6-9] based on the reasonable hypothesis that dystocia is partly due to lactic acidosis of myometrial tissue [10]. Recent physiological studies on the effects of acidification of the uterus show human myometrium to be sensitive to changes in pH, and that accumulation of lactic acid in myometrium during contractions may reduce the strength of contractions [7,11]. More recent studies of the acid–base balance of uterine tissue indicate that MCT 4 proteins, which appear to be activated when the myometrium is hypoxic, may be the mechanism by which lactic acid in myometrium is transported to the surrounding amniotic fluid [12].

If amniotic fluid lactate is relevant in the clinical management of dystocia, it is essential that a rapid and accurate test for amniotic fluid lactate is available. Although measurement of lactate using laboratory based spectrophotometric and fluorometric assays is possible this is not clinically useful in diagnosing dystocia due to time constraints in the clinical setting of active labour. The Lactate Pro is a small, portable hand held device used to process blood samples at the point of care which is relatively inexpensive. It has the potential to be used in rural and remote settings where laboratory technology may be unavailable due to costs and /or operator skill and has been found to be easy to operate and accurate [13,14]. Manufacturer’s specifications indicate the Lactate Pro meter measures lactate concentrations in whole blood between 0.8 and 23 mmol/L and previous tests of reliability in amniotic fluid show minimal measurement error with a coefficient of variation of (1.7–3.0%) [15]. However, validation studies for use of the Lactate Pro to test lactate in amniotic fluid are not well described, and previous studies of amniotic fluid lactate not sufficiently powered to determine the utility of amniotic fluid lactate measurement for women in labour.

This report describes an assessment of a handheld meter (the Lactate Pro) for measuring amniotic fluid lactate, prior to conducting a large prospective study. The primary objectives of the pilot amniotic fluid lactate study were to assess the consenting processes for recruiting women to amniotic fluid lactate research and evaluate methods of amniotic fluid collection. We also aimed to determine the clinical aspects of relevant confounding factors in the measurement of lactate in amniotic fluid by contaminants, and determine the concordance between bedside measurement of lactate using the Lactate pro hand held meter and reference laboratory biochemical measurements.

## Methods

### Pilot study population and eligibility criteria

Women who booked to give birth at a tertiary teaching hospital between October 2011 and January 2012 were considered for inclusion. Nulliparous women with a live, singleton cephalic fetus and a spontaneous onset or induction of labour at 37–42 weeks gestation were eligible to participate. A final decision regarding inclusion was made when the woman was in labour to ensure the fetus remained in a cephalic presentation and was not compromised, as evidenced by routine admission cardiotocography. Women were excluded if they had a known fetal anomaly or a recognised contraindication to vaginal birth at the onset of labour. Three women with a singleton fetus with polyhydramnios undergoing elective amniocentesis in the third trimester of pregnancy were also recruited, in order to collect amniotic fluid under sterile conditions. The Study Protocol was approved by New South Wales Northern Network Human Research Ethics Committee 11/140 through the National Ethics Application Form Submission AU/19E1A08.

### Recruitment, consent and enrolment

Recruitment took place at antenatal visits at or after 36 weeks gestation at the Outpatient Department of a public tertiary level teaching hospital. When a woman who had consented to participate in the study arrived at hospital in labour, or for an induction, the research midwife was notified by staff. The research midwife confirmed that all inclusion criteria and none of the exclusion criteria were present and the woman was enrolled into the study, after giving verbal confirmation of her consent to participate.

### Sample collection and amniotic fluid testing in the birthing room

Amniotic fluid samples were collected from labouring women at the following defined time points after arrival at the hospital. On admission if membranes were already ruptured; at spontaneous rupture of membranes during labour; at the time of artificial rupture of membranes; at routine vaginal assessment of labour progress (usually four hourly); prior to augmentation of labour; and at the time of birth. Amniotic fluid was not collected if a woman had not ruptured her membranes.

Non-invasive methods for collecting amniotic fluid were trialled by the research midwife. It became apparent that amniotic fluid was most easily collected at the time of vaginal examination as the fluid pooled in the cupped, gloved, palm of the examining clinician. Results were obtained either by touching the primed Lactate Pro test strip directly to the pooling fluid during the examination or, by collecting some of the pooling fluid in a needle-less syringe. Alternatively, where women had copious flowing amniotic fluid, lactate was measured by applying the primed test strip directly to a freshly applied perineal pad. In collections where a syringe was used, if sufficient fluid was available, after testing with the Lactate Pro, a portion of the sample was sent to the laboratory for reference testing. In contrast, studies by Wiberg Itzel et al. (2008) obtained amniotic fluid by inserting a silicon catheter into the labouring uterus, via the vagina. We did not adopt or test this method of obtaining amniotic fluid as we aim to determine if amniotic fluid lactate, obtained at the time of routine examination by non-invasive methods, is a potential biomarker for clinicians to assess progress of labour.

Amniotic fluid specimens were tested at the time of collection using the Lactate Pro analyser according to manufacturer’s instructions. Briefly, a test strip was inserted into the Lactate Pro, ensuring correct orientation. The device, with test strip in situ was dipped in a vertical position into the amniotic fluid sample. Results, available within 1 minute were recorded by the research midwife. In most cases, clinical staff remained blinded to the result, except for teaching purposes where the research midwife shared the results with clinical staff. Amniotic fluid lactate results were never considered in clinical decision making.

### Validation of point of care amniotic fluid lactate measurement

Amniotic fluid lactate results were compared to validated reference laboratory techniques for measuring lactate in aqueous fluids, the ABX Pentra 400 (Horiba Medical) using the following methods.

At the time of point of care testing with the Lactate Pro, where sufficient amniotic fluid sample was available, amniotic fluid was collected via syringe and inserted into sodium fluoride preservative tubes (Becton Dickinson). Within one hour of collection amniotic fluid samples were centrifuged at 8000×*g* for 2 minutes. The supernatant was removed and stored at −20°C until analysis by the ABX Pentra 400, courtesy of the Clinical Chemistry Laboratory at South Eastern Area Laboratory Services, Randwick.

In order to determine if the sodium fluoride preservative used in sample preparation for the AbX Pentra 400 affected lactate levels, a minimum of 0.6 mL of saline was added to the sodium fluoride tubes, then spiked with lactate substrate (62 mmol/L) from the LDH12 kit (Roche, SUI) to yield estimated low, high and midrange values. The selected values were based on previous lactate studies [8,15,16]. Saline samples without fluoride preservative containing the same concentration of lactate were also prepared. Each sample was prepared in triplicate and measured using the Lactate Pro device (Table 1).

**Table 1:** Triplicate saline samples spiked for predicted Lo, Med and Hi results

	**No lactate**	**‘Low’ lactate**	**‘Medium’ lactate**	**‘High’ lactate**	**Lactate 62 mmol/L**
Saline	Lo*	1.5 ± 0.2	7.0 ± 0.1	21.1 ± 0.2	Hi
Saline + fluoride preservative	Lo*	1.6 ± 0.6	6.8 ± 0.1	19.8 ± 0.2	Hi*

*Manufacturers instructions for the Lactate Pro indicate The Lactate Pro will display “Lo” when lactate levels are below 0.8 mmol/L and “Hi” will be displayed when lactate > 23.3 mmol.

Samples of amniotic fluid collected under sterile conditions were spiked with lactate and levels measured using both the Lactate Pro and AbX Pentra 400. Baseline biological lactate concentration was determined by testing the amniotic fluid samples with the Lactate Pro meter. A minimum of 0.6 mL of amniotic fluid was then spiked with lactate to yield a predetermined lactate concentration (0.5 mM, 1.0 mM, 5 mM, 10 mM, 20 mM) above baseline. Each concentration was prepared and tested in triplicate. At the time of spiking, samples were tested using the Lactate Pro meter, and the sample centrifuged and stored until laboratory analysis by the AbX Pentra, as described above (Table 2).

**Table 2:** A comparison between Lactate Pro meter results and the automated lactate analyser (Abx Pentra 400) of amniotic fluid collected under sterile conditions and spiked with a known quantity of lactate

**Lactate concentration**	**[Lactate] (mmol/L) Lactate Pro**	**[Lactate] (mmol/L) AbX Pentra 400**
*Baseline*	9.5 ± 0.1	10.0 ± 0.2
0.5 mM*	10.0 ± 0.1	10.6 ± 0.5
1.0 mM	10.5 ± 0.5	10.5 ± 0.1
5 mM	14.6 ± 0.6	14.2 ± 0.3
10 mM	21 ± 0.8	18.4 ± 0.1
20 mM	Hi	26.3 ± 0.7

* Lactate concentration added (in addition to baseline levels of lactate in amniotic fluid).

To assess the effect of potential amniotic fluid contaminants (meconium, obstetric cream surgical lubricant and blood) which may confound amniotic fluid lactate results, samples of amniotic fluid were prepared containing 1%, 5%, 10% and 20% [weight per volume (w/v) or volume per volume v/v)] of the potential contaminants. The samples were tested for baseline lactate concentration and prepared in triplicate. Glove lubricant was not tested at 20% (v/v) as the mixture was too viscous for analysis by the Lactate Pro (Table 3).

**Table 3:** Assessment of variations in lactate concentration using the Lactate Pro in the presence of common obstetric contaminants

**Additive to amniotic fluid**^**#**^	**Concentration of additive**	**[Lactate] (mmol/L) Lactate Pro**
Glove lubricant*	Baseline	11.0
	1% (v/v)	11.0
	5% (v/v)	10.8
	10% (v/v)	10.1
Blood	Baseline	6.3 ± 0.9
	1% (v/v)	5.9 ± 0.3
	5% (v/v)	6.1 ± 0.3
	10% (v/v)	6.4 ± 0.4
	20% (v/v)	7.9 ± 0.5
Obstetric cream	Baseline	6.3 ± 0.9
	1% (v/v)	6.4 ± 0.5
	5% (v/v)	6.0 ± 0.5
	10% (v/v)	5.9 ± 0.3
	20% (v/v)	5.0 ± 0.1
Meconium	Baseline	7.8 ± 0.0
	1% (w/v)	7.7 ± 0.3
	5% (w/v)	7.4 ± 0.2
	10% (w/v)	7.5 ± 0.3
	20% (w/v)	7.7 ± 0.3

^#^ Amniotic fluid was thawed from–20°C prior to use, except for glove lubricant where amniotic fluid was fresh.

* Glove lubricant was not tested in triplicate and the reading was taken after 30 min.

As unintended delays may occur between the collection and testing of samples in the clinical environment, we performed a time series to determine the effect of time delays (on lactate concentration). When sufficient quantity of sample was available, after ascertaining baseline amniotic fluid lactate at point of care, a drop of the sample remaining in the collecting syringe was re-tested after 5, 15 and 30 minutes to identify possible degradation of lactate. Lactate stability studies were extended to 24 hours after collection of two amniotic fluid samples collected under sterile conditions (one spiked with 10 mmol/L lactate and one sample unspiked); the spiked, non-centrifuged amniotic fluid samples were stored overnight at 4°C, then tested after 1, 5, 15 and 30 minutes at room temperature.

## Results

### Validation of lactate Pro as an accurate measure of amniotic fluid lactate

The lactate concentrations in saline samples with and without fluoride preservative were almost identical (Table 1). While average lactate concentrations measured were similar, there was more variation in the samples with preservative. This may have been due to a problem of homogenization of the sample with the sodium fluoride crystals, most likely a preanalytical problem than an analytical variation of the device at low concentrations.

**Figure 1:**
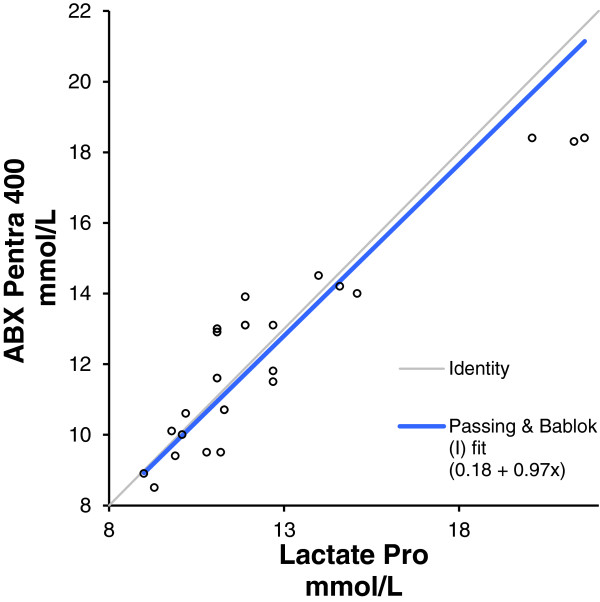
Scatter plot with passing and Bablock Fit comparing Lactate Pro and AbX amniotic fluid lactate results.

There was close concordance between results of lactate levels on the two analysis systems (Lactate Pro, AbX Pentra 400). Minor variation was apparent; mostly at high lactate concentration (≥ 10 mmol/L), which are at the upper end of the Lactate Pro analysis range as the Lactate Pro has a narrower analysis range than the AbX Pentra (Table 2). A Passing & Bablok regression line fitted to the results found the Lactate Pro to be accurate from 9–20 mmol/L, with y = 0.18 + 0.97x (95% CI 0.76–1.45) (Figure 1).

### Assessment of potential confounders of lactate levels in amniotic fluid

Amniotic fluid lactate increased in the presence of whole blood (which has its own lactate load) and decreased in the presence of other contaminants as the ratio of contaminant to amniotic fluid increased (Table 3). In the time series analysis, samples collected during labour and under surgical conditions indicated that lactate concentrations remained stable with no significant reduction in measured lactate up to 30 min after initial collection, and remained stable in samples refrigerated overnight and tested at least 24 hours after collection (data not shown).

In total, 35 women were recruited to the study and 90 attempts were made to collect amniotic fluid from 20 labouring women who gave birth during the study timeframe. Amniotic fluid was not able to be collected from 15 women as nine arrived in hospital in advanced labour, three had reduced liquor volume diagnosed by ultrasound, two had not given birth at the time of data analysis and one woman gave birth with intact membranes. A lactate result was measured in 65/90 attempts to collect amniotic fluid. Mucous samples appeared to prevent the capillary action of the Lactate Pro test strip and no results were obtained from mucousy samples. Nor were results obtained at the time of artificial rupture of membranes performed to induce labour on 10 occasions; despite the procedure, sufficient amniotic fluid for sampling was not detectable at that time. Several other factors impacted on successful readings for example, one ‘Lo’ result was obtained from a sample collected directly after a woman exited the bath (presumably bathwater contamination). Two ‘Lo’ results were attributable to operator error (failure to prime the test strip by inserting it into the meter prior to collecting the sample). Outlier ‘Lo’ results were removed from the analysis.

## Discussion

Results show the handheld Lactate Pro meter to be reliable measuring lactate in amniotic fluid samples collected and tested in controlled conditions. A Passing & Bablok regression line applied to the lactate measures shows the Lactate Pro meter accurate from 9–20 mmol/ when measuring lactate in amniotic fluid collected under sterile conditions, compared to results from the reference analyser. A Passing Bablok regression was chosen due to the small sample size, as it allows measurement error (imprecision) in both the X and Y variables, does not assume measurement error is normally distributed, and is robust against outliers. The result compares favourably with correlation reported by Tanner et al. [14] comparing the accuracy of the Lactate Pro against a laboratory-based a Radiometer ABL 700 analyser.

Our results support previous findings by others who have assessed the Lactate Pro in amniotic fluid lactate research [8,9,15] although we clearly outline the potential effects of contaminants on lactate levels and describe collection methods that reflect clinical practice, not previously described to our knowledge. Reassuringly, we established that possible contaminating substances encountered during labour do not alter amniotic fluid lactate results to any great extent and our small time series leads us to conclude that delays encountered in the clinical situation, prior to testing, do not unduly affect results*.*

Although the Amniotic Fluid Lactate™ monitoring system is now available on the market prior to conducting clinical trials to demonstrate the efficacy of amniotic fluid lactate in obstetric clinical decision making [17], we remain interested in exploring the utility of handheld meters to test amniotic fluid lactate for the reasons of cost efficiency, potential use in remote settings and versatility, as handheld lactate members have also been clinically evaluated to test fetal scalp and umbilical cord lactate [18,19].

## Conclusions

This small pilot study establishes proof of principle that the Lactate Pro meter, a commercially available product validated to measure lactate in whole blood, is a reliable measure of lactate in amniotic fluid, in samples collected from women during labour after spontaneous or artificial rupture of membranes. Moreover, we found women interested and willing to participate in amniotic fluid lactate research, although it will be necessary to reduce selection and sampling bias in future amniotic fluid lactate research studies, in order to achieve valid results about the association of lactate results with labour dystocia.

## Competing interests

All authors declare that they have no competing interests.

## Authors’ contributions

All the authors had full access to all of the data (including statistical reports and tables) in the study. BH and SKT initiated the study, participated in its coordination, drafted the manuscript and gave final approval of the version to be published. BH, WR & JI designed the study protocol, BH obtained ethical approval, recruited women, collected specimens and JI & BH performed sample validation studies and drafted the manuscript. MM conducted reference laboratory tests. JI and MBT performed statistical analysis. All authors commented on and approved the final manuscript.

## References

[B1] Lowe N A review of factors associated with dystocia and caserean section in nulliparous women Journal of Midwifery Womens Health 2007 52 3 216 228 10.1016/j.jmwh.2007.03.003 17467588

[B2] Li Z McNally L Hilder L Sullivan E Australia’s Mothers and babies Perinatal statistics series 2009 Sydney: Australian Institute of Health and Werlfare

[B3] Kjaergaard H Olsen J Ottesen B Nyberg P Dykes A-K Obstetric risk indicators for labour dystocia in nulliparous women: a multi-centre cohort study BMC Pregnancy Childbirth 2008 8 1 45 10.1186/1471-2393-8-45 18837972 PMC2569907

[B4] National Institute for Health and Clinical Excellence Intrapartum care: care of healthy women and their babies during childbirth *NICE clinical guidelines.* vol. 55 2007 National Institute for Health and Clinical Excellence Accessed March 25, 2012 http://publications.nice.org.uk/intrapartum-care-cg55/guidance 25950072

[B5] Neal JL Lowe NK Patrick TE Cabbage LA Corwin EJ What is the slowest-Yet-normal cervical dilation rate among nulliparous women with spontaneous labor onset J Obstet Gynecol Neonatal Nurs 2010 39 361 369 10.1111/j.1552-6909.2010.01154.x 20629924 PMC2928658

[B6] Akerud H Ronquist G Wiberg-Itzel E Lactate distributionin culture medium of human myometrial biopsies incubated under different conditions Am J Physiol Endocrinol Metab 2009 297 E 1414 1419 10.1152/ajpendo.00458.2009 19826101

[B7] Wray S Insights into the uterus Exp Physiol 2007 92 4 621 631 10.1113/expphysiol.2007.038125 17468199

[B8] Wiberg-Itzel E Pettersson H Andolf E Hansson A Winbladh B Akerud H Lactate concentration in amniotic fluid: a good predictor of labor outcome Eur J Obstet Gynecol Reprod Biol 2010 152 34 38 10.1016/j.ejogrb.2010.05.005 20542626

[B9] Wiberg-Itzel E Pettersson H Cnattingius S Nordstrom L Association between lactate concentration in amniotic fluid and dysfunctional labor Acta Obstetricia et Gynecologica Scand 2008 87 9 924 928 10.1080/00016340802295636 18720033

[B10] Quenby S Pierce S Brigham S Wray S Dysfunctional labour and myometrial lactic acidosis Obstet Gynaecol 2004 104 4 718 723 10.1097/01.AOG.0000118306.82556.43 15051564

[B11] Taggart M Wray S Hypoxia and smooth muscle function: key regulatory events during metabolic stress J Physiol 1998 509 313 325 10.1111/j.1469-7793.1998.315bn.x PMC2230985 9575282

[B12] Ullah M Davies A Halestrap A The plasma membrane lactate transporter MCT 4, but Not MCT1, is up regulated by hypoxi through a HIF-1_ dependent mechanism Joural of Biology and Chemistry 2006 281 14 9030 9037 10.1074/jbc.M511397200 16452478

[B13] Pyne D Boston T Martin D Logan A Evaluation of the Lactate Pro blood lactate analyser European Journal Of Applied Physiology 2000 82 1–2 112 116 10879451 10.1007/s004210050659

[B14] Tanner R Fuller K Ross M Evaluation of three portable blood lactate analysers: lactate Pro, lactate scout and lactate plus European Journal Of Applied Physiology 2010 109 3 551 559 10.1007/s00421-010-1379-9 20145946

[B15] Wiberg-Itzel E Cnattingius S Nordstrom L Lactate determination in vaginal fluids: a new method in the diagnosis of prelabour rupture of membranes BJOG 2005 112 6 754 758 10.1111/j.1471-0528.2004.00521.x 15924532

[B16] Wiberg-Itzel E Lipponer C Norman M Herbst A Prebensen D Hansson A Bryngelsson AL Christoffersson M Sennstrom M Wennerholm UB Determination of pH or lactate in fetal scalp blood in management of intrapartum fetal distress: randomised controlled multicentre trial BMJ 2008 336 7656 1284 1287 10.1136/bmj.39553.406991.25 18503103 PMC2413392

[B17] ObsteCare The solution for dysfunctional labour Accessed March 25, 2013 http://www.obstecare.com/index.html

[B18] Nordstrom L Lactate measurements in scalp and cord arterial blood Current Opinion in Obstetrics and Gynaecology 2001 13 141 145 10.1097/00001703-200104000-00008 11315868

[B19] Pennell CE Tracy MB Donald MC Accurate lactate-a new method for rapid pont of sample measurement of lactate in fetal and neonatal blood Neonatal Intensive Care 2000 13 4 53 58

